# Role of Sirtuins in Diabetes and Age-Related Processes

**DOI:** 10.7759/cureus.28774

**Published:** 2022-09-04

**Authors:** Nimisha Lingappa, Harvey N Mayrovitz

**Affiliations:** 1 Osteopathic Medicine, Nova Southeastern University Dr. Kiran C. Patel College of Osteopathic Medicine, Davie, USA; 2 Medical Education, Nova Southeastern University Dr. Kiran C. Patel College of Allopathic Medicine, Davie, USA

**Keywords:** aging, type 2 diabetes mellitus, sirtuin proteins, caloric restriction, intermittent fasting

## Abstract

The practice of intermittent fasting continues to grow as a widely practiced diet trend due to its feasibility and reported high success rate. By practicing intermittent fasting, levels of sirtuin proteins (SIRTs), also known as the longevity protein, rise in the body and bring numerous health benefits. Currently, seven SIRTs have been described in humans in different locations of the cell with a wide variety of corresponding functions including gene transcription, DNA repair, and protection against oxidative damage. SIRT activators, such as resveratrol found in red wine, are also commonly consumed to amplify the health benefits associated with protection against diabetes and age-related disease processes. The purpose of this review is to explore the interaction of intermittent fasting on SIRT levels and how the increase in these proteins impacts age-related disease processes. The understanding of SIRTs is continuously evolving as more interactions and SIRT-specific activators are being revealed. New discoveries are crucial for forming potential therapeutics that delay many common diseases and promote healthy living.

## Introduction and background

Intermittent fasting is a rapidly growing health practice worldwide that consists of alternating periods of eating and fasting done on a regular basis [1]. According to the International Food Information Council’s (IFIC) Food and Health Survey, 43% of Americans claim to be following a specific diet or eating pattern and 10% of those individuals follow intermittent fasting, making it the most popular diet [2]. Intermittent fasting is not a traditional type of diet in which food intake is limited, but instead controls the timing of food consumption. Common fasting regimens include alternate day fasting, fasting two out of seven non-contiguous days of the week, and eating during limited time intervals of each day [3]. With decreased energy intake, the body continues to consume glucose from glycogen stores. As glycogen stores are depleted, the mobilization of fatty acids and the creation of ketone bodies begins [3]. These processes trigger an increase in growth hormone and a decrease in insulin that results in the promotion of fat usage and loss to create more efficacious energy.

One of the main reasons for the rise in popularity of intermittent fasting is the feasibility of following such a program. For instance, limiting food intake to eight hours a day is doable even with a busy schedule. Similar to the ways of hunter-gatherers facing periods of food shortage, fasting promotes evolutionarily conserved adaptations for increasing weight loss, decreasing insulin resistance, and preventing age-related processes and diseases [1]. During periods of feeding, cells engage in growth processes regardless of cell conditions. However, during fasting, cell pathways are activated to defend against meal-induced oxidative and metabolic stress and promote tissue repair [3]. 

Aside from the postulated health benefits of intermittent fasting, certain forms of caloric restriction cause an increase in sirtuin proteins. Sirtuin proteins (SIRTs) are a class of nicotinamide adenine dinucleotide (NAD) dependent lysine-specific deacetylases and represent homologs of yeast silent information regulator (SIR2) [4]. These class three histone deacetylases are enzymes that remove an acetyl group from N-acetyl-lysine residues on protein substrates [5]. Currently, seven isoforms of SIRTs exist in mammals. SIRT1, SIRT6, and SIRT7 are localized to the nucleus, SIRT2 is cytoplasmic, and SIRT3, SIRT4, and SIRT5 are mitochondrial proteins [5]. Since SIRTs are located throughout the cells, a wide variety of cell functions exist. Some of these physiological functions include regulation of healthy aging, genome stability, mitochondrial physiology and biogenesis, and cellular metabolism [4]. The wide expanse of SIRT functions also leads to implications for certain pathological processes and possible novel targets for therapy. SIRT activation can be useful against aging-related disorders of metabolism, cardiovascular and neurodegenerative diseases, and other vascular processes. On the other hand, SIRT inhibition can be of use in controlling the progression of cancer, HIV, and other muscular diseases [6]. Some of these effects will be further explored in this review. 

As previously mentioned, intermittent fasting may help alleviate obesity, insulin resistance, inflammation, and other age-related processes. Specifically, intermittent fasting has been reported to reverse insulin resistance in people with type 2 diabetes mellitus [3]. Caloric restriction leads to lower levels of insulin-like growth factor, growth hormone, and inflammatory markers that are in part responsible for the pathogenesis of type 2 diabetes [3]. One study in 2003 reported significant reductions in percent body fat, hemoglobin A1c (HbA1c), and triglyceride levels from intermittent fasting [7]. By measuring various health markers at baseline and after periods of intermittent fasting, beneficial effects have been reported in the form of increased cell and tissue resistance against common diseases associated with aging, sedentary lifestyles, and overconsumption [1].

Many SIRT activators currently exist and are in use for their health benefits such as green tea, turmeric, and kale. Currently, SIRT1 is the most studied isoform for its role in caloric restriction and as a target in preventing age-related diseases [6]. Resveratrol, a plant polyphenol made in response to infections, also acts as an activator of SIRT1 [8]. Dietary sources of resveratrol can be found in grapes, red wine, and peanuts. Resveratrol has been reported to delay age-related diseases through its actions as a SIRT1 activator, and its use and features have become a popular field of interest in recent years [9]. 

## Review

This review will explore and detail the vast effects of intermittent fasting and caloric restriction mimetics such as resveratrol and other SIRT activators as they relate to the pathogenesis of age-related processes and their potential as useful therapeutics. The specific aim of this review is to elucidate the role of sirtuin proteins on various disease processes including type 2 diabetes mellitus and certain forms of cancer. The goal is to clarify the interaction between intermittent fasting resulting in an increase in sirtuin proteins throughout the body and how these increased levels affect the progression of disease in both positive and negative ways. 

Search strategy

The PubMed database was searched for peer-reviewed articles written in English using the following criteria. Five separate searches were conducted for different areas of interest. Each search used the keyword “Sirtuin” in the title as sirtuins are of primary focus in this review. The five searches conducted were as follows: “Sirtuin” and “Diabetes” in the title, “Sirtuin” and “Fasting” in the title, “Sirtuin” and “Vascular” in the title, “Sirtuin” and “Age” in the title, and “Sirtuin” and “Review” in the title. A total of 116 papers met the keyword search criteria. References used by the resulting papers were also accessed as needed. 

Sirtuin production and bioavailability 

SIRTs are evolutionarily conserved proteins that exist in a wide variety of organisms ranging from bacteria to humans [10]. SIRTs are NAD+ dependent deacetylases and since NAD+ is needed for most enzymatic activities SIRTs may also function as metabolic sensors that affect the pathophysiology of aging [11]. Of the various precursors for NAD, nicotinamide was reported to be more effective than nicotinic acid in regulating glucose metabolism and the SIRT1 pathway [11]. Numerous experiments on multiple species including yeast, flies, and mice have indicated that deleting SIRTs removes the beneficial effects of caloric restriction resulting in reduced longevity [4]. Substrates of various processes are specific to each SIRT; the role of SIRT1 in chromatin remodeling and DNA damage response is one example [10]. Some SIRT-related functions overlap between the seven isoforms of SIRTs, such as SIRT1 and SIRT2 both regulating hepatic gluconeogenesis. Furthermore, organ systems can be grouped to study how various SIRTs affect processes. For instance, SIRT1 through SIRT6 have metabolic functions in the liver and can be further studied for the possible development of novel therapies against age-related diseases of the liver [10]. 

**Figure 1:**
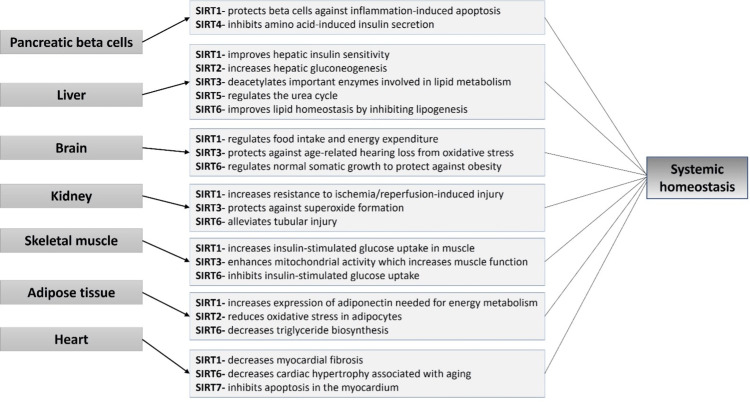
Specific Metabolic Functions of Sirtuins by Organ Sirtuin proteins (SIRTs) have specific deacetylation activities on protein substrates that are vital to metabolic processes in the mentioned organ systems. These diverse biological functions play a crucial role in DNA repair, mitochondrial biogenesis, antioxidant production, and cellular metabolism [12]. Some functions of SIRTs specific to each organ are included in the figure to provide examples of organ-specific roles. Although redundancy exists between protein substrates and SIRTs, each has a specific role in modulating oxidative stress, inflammation, and dysfunction [13]. By acting on the multifactorial processes of aging, pathological changes and progressive decline can be minimized leading to systemic homeostasis.

As shown in Figure 1, SIRTs can be categorized into specific locations by organ system throughout the body [10]. In these organ systems, SIRTs have specific deacetylation activities on protein substrates that are vital to metabolic processes. These diverse biological functions play a crucial role in DNA repair, mitochondrial biogenesis, antioxidant production, and cellular metabolism [12]. Some functions of SIRTs specific to each organ are included in the figure to provide examples of organ-specific roles. Although redundancy exists between protein substrates and SIRTs, each has a specific role in modulating oxidative stress, inflammation, and dysfunction [13]. By acting on the multifactorial processes of aging, pathological changes and progressive decline can be minimized. 

Activators of SIRT1 and related actions

Due to the numerous reported health benefits of SIRTs, research has been directed toward the discovery of SIRT activators, with activators of SIRT1 being the most studied. One such activator is resveratrol, a small polyphenol, which can increase SIRT1 activity 13-fold resulting in beneficial effects on cataracts, bone structure, vascular health, and locomotor activity leading to improvements in general health [4]. One of the most abundant natural sources of resveratrol is from grape seeds of *Vitis vinifera*, the common grape vine used to make wine [4]. To test resveratrol’s impact on energy metabolism and mitochondrial function, 11 healthy, obese men consumed 150 mg of resveratrol per day for 30 days [14]. In this study, resveratrol was reported to activate SIRT1, decrease triglyceride levels, and improve muscle mitochondrial respiration. These metabolic changes mimic the effects of caloric restriction associated with intermittent fasting. Resveratrol can be absorbed in the gastrointestinal tract and distributed throughout the body to prevent cardiovascular disease, protect pancreatic cells by inhibiting poly-ADP ribose polymerase, a family of proteins involved in cellular processes, and alleviate metabolic syndrome all by acting as a free radical scavenger [15].

In addition, a SIRT1 activator with 1000-fold greater potency compared to resveratrol called SRT1720 has been synthesized and reported to be efficacious against type 2 diabetes mellitus and cancer in mice [4]. SRT1720 was also reported to improve cognitive function in mice with type 2 diabetes mellitus along with increasing body weight, reducing fasting blood glucose, and minimizing proteins associated with oxidative stress and inflammatory damage [16]. Much is yet to be learned regarding SIRTs and their various roles.

SIRT1 and age-related chronic conditions

When studying the pathogenesis of age-related diseases in mice, different methods were used to demonstrate the effects of a deletion of SIRT1 in various organs. With the utilization of myeloid cell-specific SIRT1 knockout mice, deletion of SIRT1 resulted in increased transcription of proinflammatory target genes, reactive oxygen species, and an amplified inflammatory response [17]. These changes mimic those experienced in many chronic inflammatory diseases and predispose the mice to developing insulin resistance and metabolic disorders. An additional result of SIRT1 deletion is an increase in nuclear factor kappa beta (NF-kB) dependent proinflammatory cytokines such as interleukin-1 (IL-1), tumor necrosis factor-alpha (TNF-alpha), and many others [18]. The potential importance of such changes is exemplified by considering the role of NF-kB in regulating inflammation involved in non-alcoholic fatty liver disease and non-alcoholic steatohepatitis [19]. Overexpression of SIRT1 in the liver helps decrease these proinflammatory cytokines by downregulating NF-kB, but deletion of SIRT1 exacerbates hepatic inflammation [19]. A similar trend of different transcription factors affecting disease processes can be seen with pancreatic and duodenal homeobox factor 1 (PDX1) and Forkhead box class O (FOXO). PDX1 plays an important role in pancreatic development and beta cell differentiation, and mutation in the PDX1 gene can cause maturity-onset diabetes in the young [19]. SIRT1 expression in beta cells helps enhance insulin production to manage glucose levels and prevent the onset of diabetes. SIRT1 regulates FOXO transcription, and FOXO controls biological responses to prevent neurodegeneration in response to oxidative stress [18]. Specifically, FOXO prevents the accumulation of alpha-synuclein in the brain and age-mediated memory deficits [19]. A number of these processes will be further elucidated in this review.

SIRT6 and diabetes

SIRT6, a sirtuin protein that localizes to the nucleus, has recently been a topic of interest due to its confounding roles in metabolic homeostasis. Since SIRT6 is found in the nucleus, it is primarily involved in modulating transcription by histone deacetylation of chromatin [20]. SIRT6 levels increase in response to nutritional stress through the regulation of SIRT1. SIRT6 is increased in the heart, kidney, brain, and white adipose tissue after periods of caloric restriction and is decreased in insulin-resistant mice and humans. Overexpression of SIRT6 in mice provides protection against metabolic pathologies from diet-induced changes, but SIRT6 knockout mice have increased glucose transport and decreased levels of TNF-alpha in the serum which has a negative effect on insulin resistance [21]. The controversy regarding activation or inhibition of SIRT6 activity as a therapeutic against type 2 diabetes mellitus stems from the idea that SIRT6 inhibition is able to prevent the repression of glucose transporters and enzymes [20]. One study administered a SIRT6 inhibitor to mice for 10 days and saw an improvement in glucose tolerance, increased expression of glucose transporters, and decreased insulin, triglycerides, and cholesterol levels in serum [22]. On the other hand, another study monitored diabetic wound healing in SIRT6 knockdown mice and found increased levels of oxidative stress, decreased angiogenesis and vascularization, and impaired wound closure [23]. With these increased levels of oxidative stress in the vasculature, SIRT6 deficiency leads to endothelial dysfunction seen in various aging processes [24]. As activators and inhibitors of specific SIRTs are still lacking, more research is needed to fully understand methods to improve glycemic control in type 2 diabetes. 

Diabetic insulin resistance

SIRT1 plays a major role in the regulation of metabolic homeostasis, and as a result, any changes causing downregulation can stimulate pathways leading to chronic complications of diabetes mellitus and its comorbidities. Specifically, during a fasting state, decreased insulin and increased glucagon stimulate gluconeogenesis. Increased caloric restriction causes a SIRT1-dependent decrease in glucose, increased catabolism of fatty acids, decreased cholesterol uptake into the intestine, and limited fatty liver disease and inflammation [25]. A link between FOXO, signal transducer and activator of transcription 3 (STAT3), and SIRT1 has been identified to regulate gluconeogenesis [26]. Specifically, SIRT1 regulates the deacetylation of STAT3, deactivating it, and preventing transcription of genes involved in gluconeogenesis in normal conditions. However, in a fasting state, SIRT1 triggers deacetylation of FOXO and peroxisome proliferator-activated receptor-gamma coactivator-1 alpha (PGC1a) which induces gluconeogenesis and inhibits glycolysis in the liver. These changes reflect the beginning stages of type 2 diabetes mellitus with insulin resistance and hyperglycemia. 

Diabetic medications and sirtuins

Many commonly used agents for the treatment of type 2 diabetes mellitus can alter SIRT1 levels and activity. For example, research suggests that SIRT1 mediates metformin’s effects in suppressing hepatic gluconeogenesis, activating fatty acid oxidation, and preventing hyperglycemia-induced retinal damage [10]. Furthermore, metformin used with caloric restriction, such as intermittent fasting, increased the expression of SIRT1 and furthered these beneficial effects [25]. Incretin mimetics, also known as glucagon-like peptide-1 (GLP-1) agonists, and dipeptidyl peptidase-4 (DPP4) inhibitors are both common classes of drugs used in the treatment of diabetes. Both stimulate pathways leading to upregulation of SIRT1 to limit oxidative stress and alleviate the progression of insulin resistance [25]. Sodium-glucose co-transporter 2 (SGLT2) inhibitors are another class of medications used in type 2 diabetes mellitus. This class of drugs has dual effects on slowing the progressive loss of renal function in those with diabetes. Compared to both GLP-1 agonists and DPP4 inhibitors, SGLT2 inhibitors are accompanied by comparable decreases in blood glucose but increased benefit in maintaining glomerular function and preventing adverse renal outcomes [27]. Specifically, SGLT2 inhibitors trigger a fasting-like state that induces both SIRT1 and adenosine monophosphate-activated protein kinase (AMPK) activation. SIRT1 and AMPK decrease cellular stress reducing possible glomerular and tubular injury seen in diabetic chronic kidney disease. 

Another drug class commonly used to treat diabetes is thiazolidinediones, a class of oral hypoglycemics that promote adipogenesis and fatty acid uptake. One agent in this class is pioglitazone. A study was conducted on 44 obese, postmenopausal females who had or did not have type 2 diabetes to investigate the effects of fenofibrate, a medication to lower cholesterol, alone or in conjunction with pioglitazone on SIRT1 levels [28]. Fasting glucose, HbA1C, serum lipids, and SIRT1 were all measured following eight weeks of a protocol of fenofibrate alone or fenofibrate with pioglitazone. All treatment groups reported an increase in SIRT1 and decreases in inflammatory markers such as interleukin 6 (IL-6) [28]. These changes produce a beneficial effect in decreasing chronic inflammation associated with type 2 diabetes and obesity. 

Diabetic renal complications

Diabetic nephropathy is one of the main complications associated with diabetes mellitus and accounts for many cases of end-stage renal disease [29]. Metabolic derangements associated with diabetes can lead to chronic low-grade inflammation from cytokines such as TNF-alpha, IL-1, and IL-6. Periods of caloric restriction can activate nutrient-sensing pathways like AMPK and SIRT1 to induce autophagy to protect against these deleterious processes in the body. However, in diabetic mice, AMPK levels are suppressed due to hyperglycemic conditions, and autophagy does not occur [30]. The suppression of AMPK triggers lipogenesis and further lipotoxicity associated with diabetic kidney disease. When metformin, a SIRT1 activator of the AMPK pathway is given, effects on glomeruli can be reversed. SIRT1 is expressed in kidney podocytes but its function is unclear. Mice with knockdown of renal SIRT1 expression had normal glomerular function under normal basal metabolic conditions [31]. However, diabetic mice with renal SIRT1 knockdown developed more albuminuria and mitochondrial dysfunction than diabetic mice with SIRT1 intact. This indicates that SIRT1 in podocytes plays a role in maintaining homeostasis under conditions of mitochondrial stress. Although some renal changes can be alleviated, long-standing diabetes and concurrent diabetic nephropathy are currently lacking effective treatment. 

Honokiol, a natural biphenolic compound isolated from magnolia bark, activates SIRT3 and acts as an antioxidant and anti-inflammatory with reno-protective effects [32]. Honokiol administration to mice activated SIRT3 which limited podocyte damage and the progression of nephropathy, but it did not activate SIRT1 or SIRT6. Selective activation of SIRT3 also provided protection against albuminuria and glomerular lesions. Other studies have demonstrated further effects of honokiol in alleviating cardiac hypertrophy and neurodegenerative disorders [33]. In addition, soluble epoxide hydrolase, an enzyme with both hydrolase and phosphatase activities that metabolize anti-inflammatory acids, can be inhibited to prevent vascular calcification in chronic kidney disease [34]. Mice with genetic deletion of soluble epoxide hydrolase had preserved SIRT3 expression which prevented vascular calcification associated with chronic kidney disease.

Diabetic neurodegenerative complications

As life expectancy continues to increase, the aging population has a proportional increase. With increasing age, the incidence of dementia continues to rise. Alzheimer’s disease is the main type of dementia and is characterized by severe cognitive impairment over time. SIRT1 is ubiquitously expressed in the brain and regulates many processes that are altered in the pathogenesis of Alzheimer’s disease such as action potential propagation, neurodegeneration, and mitochondrial dysfunction [35]. In mice, overexpression of SIRT1 led to decreased formation of beta-amyloid plaques characteristically seen in Alzheimer’s disease. Furthermore, upon autopsy of human brains with Alzheimer’s disease, expression of SIRT1 and SIRT3 were decreased [36]. In addition, dysregulation of SIRT1 influences neuronal plasticity and increases IL-1 levels involved in brain diseases such as multiple sclerosis [37]. Compared to insulin resistance in peripheral tissues, central insulin resistance is a recent discovery that is sometimes classified as type 3 diabetes. Peripheral insulin resistance seen in type 2 diabetes may trigger insulin resistance in the brain that is associated with neurodegenerative diseases. Insulin in the brain is not only responsible for energy metabolism but also for synaptic plasticity. Therefore, insulin plays a role in the pathophysiology of diabetes-related cognitive decline. Specifically, decreased SIRT1 was seen in the hippocampus of diabetic mice leading to lower learning and memory capabilities [38]. However, with intranasal administration of insulin, SIRT1 levels increased in the hippocampus of diabetic mice, and previous cognitive impairment was improved. SIRT1 also increased hippocampus dendritic spine length and density to improve memory and learning and prevent further cognitive decline.

SIRT3 is also highly expressed in the nervous system and plays a role in neuronal processes regulating brain function. As neurons age, mitochondrial malfunction occurs with decreased SIRT3 levels and the promotion of neurodegeneration [39]. Along with decreased SIRT3, increased levels of oxidative stress contribute to age-related hearing loss from inner ear neuron damage. However, caloric restriction can delay age-related hearing loss by decreasing reactive oxygen species and increasing glutathione via proper mitochondrial antioxidant functioning [39]. Numerous studies indicate caloric restriction from intermittent fasting increases SIRT3 in the hippocampus which maintains synaptic plasticity and improves cognition. 

Diabetic vascular complications

Many serious complications of type 2 diabetes mellitus are related to chronic hyperglycemia. Diabetic cardiomyopathy is one such example that is associated with hyperglycemic memory. Hyperglycemic memory is a phenomenon where the negative effects of hyperglycemia persist even after blood sugar levels return to controlled levels [40]. With this form of metabolic memory, epigenetic changes can continue to cause damage. SIRT1 exerts a positive effect by deacetylating histones along with other protein substrates to repress catalytic activity involved in diabetic cardiomyopathy. Concomitantly with long-standing diabetes, vascular endothelial dysfunction and ischemia-reperfusion injuries occur throughout the body. In the cardiovascular system, orexin B, a hypothalamus-derived neuropeptide that regulates food intake, the sleep-wake cycle, and glucose homeostasis, and SIRT1 were both decreased following an ischemia-reperfusion injury in diabetic mice [41]. Treatment with an orexin type 2 agonist and SIRT1 activator improved vascular function and decreased myocardial injury. Further treatment with a SIRT1 antagonist and orexin agonist eliminated all improvement and elucidated the actions of orexin as a possible activator of SIRT1 [41].

Metabolic syndrome, which consists of cardiovascular and metabolic risk factors such as obesity and insulin resistance, is associated with an increased risk of developing cardiovascular disease [42]. Arterial stiffness that is commonly seen in obese, diabetic individuals is associated with a loss of compliance to blood flow and is predictive of further complications such as hypertension, heart failure, stroke, and kidney failure. To mimic this metabolic syndrome and study the effects of SIRT1 on vascular smooth muscle cells, mice were given a high fat, high sucrose diet for two months [42]. After two months, these mice developed stiff aortic walls and increased inflammation. If a normal diet was subsequently started, these changes were reversible within four months indicating arterial stiffness related to obesity can be altered. If mice maintained the high fat, high sucrose diet but added overnight fasting to mimic caloric restriction, acute activation of SIRT1 in vascular smooth muscle cells led to an associated acute decrease in arterial stiffness compared to knockout mice without SIRT1 in vascular smooth muscle cells. Furthermore, overexpression of SIRT1 or administering resveratrol or SRT1720, both SIRT1 activators, reversed previous changes and prevented arterial stiffness [42]. In another study by the same group of researchers, the effects of SIRT1 in vascular smooth muscle cells in response to angiotensin 2, a potent oxidant and inflammatory stimulus, were studied [43]. Mice lacking SIRT1 in vascular smooth muscle cells had disorganized aortic walls with increased aortic stiffness and oxidant production. SIRT1 activator administration was able to suppress the oxidant-induced damage to the aorta. In addition, SIRT1 activators provide a potential therapeutic approach to prevent aortic dissection in people at risk such as those with hypertension or genetic disorders like Marfan’s syndrome [43].

Age-related vascular conditions

Aging is one of the main risk factors associated with cardiovascular disease. Deterioration is linked to lifestyle stressors along with increased inflammation and oxidative stress within the body. SIRT1 exerts cardioprotective effects by decreasing oxidative stress and improving mitochondrial function [13]. Age-related changes of increased vascular stiffness and oxidative stress make blood vessels more susceptible to vascular diseases like atherosclerosis and increase the risk of progression to abdominal aortic aneurysm. Activation of SIRT1 serves to limit vascular endothelial cell and smooth muscle cell senescence [44]. In this same study, six human abdominal aortic aneurysm samples, taken during open surgical repair, had decreased SIRT1 activity and expression. Another study obtained samples from human vessels with and without plaques undergoing carotid endarterectomy or coronary artery bypass grafting or valve replacement respectively [45]. SIRT1 expression was decreased in samples with plaques leading to increased DNA double-strand breaks and decreased subsequent DNA repair. On the other hand, overexpression of SIRT1 in endothelial cells increases nitric oxide leading to vasodilation and anti-inflammatory actions against the progression of atherosclerosis [46]. This leads to a decrease in calcification in vascular smooth muscle cells. SIRT1 activators can also upregulate endothelial cell nitric oxide synthase to increase nitric oxide and cause vasodilation. Therefore, SIRT1 activators have been proposed as therapeutic strategies against vascular calcification. 

The factors of older age, diabetes mellitus, hypertension, hyperlipidemia, cardiovascular disease, coagulopathy, and others are all common risk factors for many other vascular conditions throughout the body. In varied vascular conditions, a commonality of low SIRT levels is seen. For instance, retinal vein occlusion, one of the most common types of retinal disease leading to possible permanent vision loss, is characterized by decreased SIRT1 and increased reactive oxygen species [47]. Lower levels of SIRT1 are also associated with age-related cataracts, macular degeneration, diabetic retinopathy, glaucoma, and many others [48]. SIRT1 plays a protective role against pathological processes in ocular diseases involving inflammation, oxidative stress, and neurodegeneration. In a mouse study, administration of resveratrol enhanced SIRT1 expression and protected against retinal degeneration [49]. Furthermore, hemorrhagic shock, a severe, life-threatening complication of multiple organ failure, is associated with mitochondrial dysfunction and decreased SIRT1 and SIRT3 levels [50]. However, administration of a SIRT1 activator, can restore SIRT1 activity and improve SIRT3 activity to protect against mitochondrial injury and improve survival. 

Although most SIRT activators studied are SIRT1 activators, apelin gene therapy is one method of upregulating SIRT3 [51]. Apelin, a bioactive peptide and endogenous ligand of G-protein coupled receptors widely expressed on cells of many organs, has numerous functions based on receptor expression in vascular endothelial cells, cardiac tissue, adipose tissue, osteoblasts, and many others. Administration of apelin gene therapy causes overexpression of SIRT3 and increased myocardial density. Both effects together alleviated diabetic cardiomyopathy in mice and reduced myocardial ischemia-reperfusion injury by decreasing reactive oxygen species. 

Cancer-related actions

One of the main controversial actions of SIRTs is the promotion of cancer via deacetylation and inactivation of proapoptotic factors that would normally provide protection [6]. SIRTs normally protect against oncogenic transformation but too much activity can have tumorigenic properties. SIRT1 is overexpressed in many tumors such as prostate, breast, lung, leukemia, and lymphoma. Liposarcoma, a locally malignant mesenchymal tumor of soft tissue, is associated with increased SIRT1 and vascular endothelial growth factor (VEGF) levels [52]. SIRT1 contributes to chromatin remodeling and VEGF promotes angiogenesis which in turn promotes the progression of cancer. Increased levels of both SIRT1 and VEGF were correlated with a poor patient prognosis in this study. 

SIRT2 has varied expressions and can act as a tumor promoter or tumor suppressor based on the cell type. It is downregulated in breast and non-small cell lung cancers but upregulated in pancreatic cancer and acute myeloid leukemia [6]. The role of SIRT3 is not fully elucidated but some evidence points to overexpression in head and neck squamous cell carcinoma. SIRT7 is upregulated in breast and thyroid cancers due to hypoacetylation of an aggressive tumor marker. On the other hand, most evidence regarding SIRT6 demonstrates action as a tumor suppressor and is downregulated in many cancers [53]. Specifically, histone deacetylation activity of SIRT6 protects against abnormal telomere metabolism which is characteristic of cancer cells. New evidence indicates inhibition of SIRT5 has the potential to suppress malignant transformation of cells and provides an area for future study of cancer therapy [54]. Overall, SIRTs play a controversial role in the progression of cancer, and more information is needed to further explain this negative factor amongst all the positive impacts against age-related processes. 

## Conclusions

In this study, we sought to understand and clarify the role of SIRTs in the aging process and their potential impact on diabetes. The overall findings suggest the following. SIRTs appear to have a positive impact on the aging process in part by limiting the negative effects of multiple factors such as inflammatory mediators and metabolic stress. Further, there appear to be positive implications of SIRTs in diabetes related to decreased insulin resistance, maintainence of renal function, and minimal cognitive impairment. Classically, SIRTs serve as longevity proteins that mediate a wide array of biological functions. SIRT activators are a newer field of study and are commonly consumed daily without even realizing it. One such example, resveratrol, is found in many foods consumed to promote good health such as red wine. Although SIRTs have numerous positive health benefits, the negative effects related to cancer progression require further study to determine the balance between both. Our evolving understanding of the mechanisms of SIRTs remains fundamental in providing potential treatment options against numerous common age-related diseases. The present review has summarized the current knowledge of SIRTs and pointed to potential emerging possibilities.
